# Determinants of Immune Tolerance Induction Outcomes in 237 Persons With Severe Hemophilia A

**DOI:** 10.1111/ejh.70269

**Published:** 2026-07-23

**Authors:** Marcus Fager Ferrari, Josefin Roslund, Eric Manderstedt, Erik Berntorp, Jan Astermark, Malmö and Hemophilia Inhibitor Genetics Study Investigators

**Affiliations:** ^1^ Department of Hematology, Oncology and Radiation Physics Skåne University Hospital Malmö Sweden; ^2^ Department of Translational Medicine Lund University Malmö Sweden; ^3^ Center for Primary Health Care Research, Department of Clinical Sciences Lund University Malmö Sweden

**Keywords:** *F8* variant, hemophilia A, immune tolerance induction therapy, inhibitors, peak inhibitor titer

## Abstract

Immune tolerance induction (ITI) to eradicate an immune response against factor VIII (FVIII) has been used in hemophilia A (HA) with various success rates, costs, and treatment burden. The present study aimed to identify markers predictive of ITI success. Data were accrued from 237 patients with severe HA (FVIII level < 1%), history of persistent FVIII inhibitors ≥ 1 BU/mL and having performed ITI. Constitutional and clinical variables associated with ITI outcome were evaluated and multivariable logistic regression models developed. The *F8* variant, age at inhibitor detection, and historical peak inhibitor titer were associated with ITI outcome. HLA DRB1*1501 and DQB1*0602 were linked to each other, but not associated with ITI outcome. In a multivariable‐adjusted model, large gene deletions, peak titer ≥ 25 BU/mL, and higher age at inhibitor detection were associated with a significantly lower ITI success rate. The discriminatory power of a predictive model was 0.87 (95% confidence interval [CI], 0.85–0.93) with the historical peak titer and 0.69 (95% CI, 0.64–0.80) without. We conclude that the peak inhibitor titer is a key determinant for ITI outcome and additional constitutional markers need to be defined for useful prediction in the clinical setting.

## 1 Introduction

Hemophilia A (HA) is a congenital bleeding disorder characterized by a deficiency of coagulation factor VIII (FVIII). For decades, the mainstay of hemophilia treatment has been replacement therapy with the infusion of FVIII concentrates [1], but when replacing the missing factor, approximately one third of persons with severe and 10%–20% of patients with nonsevere HA develop neutralizing antibodies (inhibitors) [2, 3]. These inhibitors compromise the efficacy of the infused factor, leading to increased bleeding tendency, morbidity, and management complexity.

Immune tolerance induction (ITI) therapy is the key intervention aimed at eradicating inhibitors by repeated and sustained FVIII exposure with or without the combined use of immunosuppressive agents, thereby promoting desensitization and tolerance [4, 5, 6]. The use of various ITI protocols has been reported, predominantly categorized into high‐, intermediate‐, and low‐dose regimens [7, 8]. Published data report ITI success rates ranging from 50% to 80%, with potential determinants of the response including patient age, ethnic origin, type of causative *F8* variant, time from inhibitor detection to initiation of ITI, pre‐ITI inhibitor titer, peak inhibitor titer before and during ITI, and regimen characteristics such as product type, dosing, and use of immunosuppressive agents [8, 9]. Successful ITI is defined by the sustained disappearance of inhibitors, restoration of normal FVIII pharmacokinetics, and/or the ability to resume effective replacement therapy. Nevertheless, ITI failure or inhibitor relapse remains a clinical challenge, not least in the context of a significant treatment burden and economic impact, highlighting the necessity for individualized patient stratification and vigilant monitoring. This is not least of relevance in the new therapeutic era of hemophilia management with the access of ultralong FVIII concentrates intravenously and nonfactor therapies administered subcutaneously such as emicizumab and anti‐TFPI [10]. As emerging therapies reshape treatment paradigms, ITI may continue to play a role in comprehensive hemophilia care, enabling optimization of treatment from medical, quality‐of‐life, and psychosocial perspectives. However, ITI will not be pursued in all patients and should be considered within an informed shared decision‐making framework involving patients, families, and hemophilia teams.

The Hemophilia Inhibitor Genetics Study (HIGS) was initiated in 2004 and the main outcomes of the study, focusing on genetic factors associated with inhibitor development, have been reported [11]. These data highlighted the complexity of the immune response to FVIII and suggested that multiple immune regulatory genes may be important. Consequently, identifying key genetic markers capable of consistently predicting tolerance across different cohorts has not been possible. In the present study, we focused on ITI outcomes and prediction by evaluating a combined cohort comprising HIGS participants and patients with severe HA treated at the Malmö Hemophilia Comprehensive Care Center. The impact of variables associated with ITI outcomes, including constitutional factors, such as the underlying *F8* variant, ethnicity, and HLA class II alleles, with a particular focus on alleles consistently associated with inhibitor development (HLA‐DRB1*1501 and HLA‐DQB1*0602) [12, 13, 14, 15, 16], were evaluated in the context of treatment‐related factors. Furthermore, based on preliminary data suggesting that the likelihood of translational readthrough of nonsense variants, with potential implications for FVIII tolerance, may depend on their exact location [17, 18], we investigated whether ITI outcomes are associated with the location of nonsense variants as well as frameshift‐causing small insertions and deletions, and whether this information could improve predictive models of ITI outcome.

## 2 Methods and Materials

### 2.1 Study Population

The study population consisted of persons with severe HA (defined as baseline FVIII activity < 1%) attending the Malmö hemophilia center since 1980 and persons enrolled in the HIGS cohort having performed ITI due to a persistent inhibitor ≥ 1 BU/mL. The HIGS cohort has previously been described [19] and is composed of siblings concordant for a history of inhibitors or discordant (one with an inhibitor, the other without) and a group of singletons with inhibitors enrolled between 2004 and 2010 from Europe, North America, Latin America, and South Africa. Study participation consisted of one visit, with retrospective data collection including hemophilia history, treatment‐related factors, and hospitalizations. For all persons attending the Malmö center, the *F8* variant had been characterized at the specialized Hemophilia DNA laboratory at Skåne University Hospital/Lund University according to routine clinical practice. In the HIGS cohort, unless already locally defined and clearly documented, determination of the *F8* variant was performed at the specialized laboratory at Skåne University Hospital/Lund University or at the Institute of Experimental Hematology and Transfusion Medicine, Bonn, Germany. HLA class II alleles were also characterized at the Institute in Bonn, Germany.

Ethical approval for all patients attending the Malmö hemophilia center was obtained from the Swedish Ethical Review Authority. HIGS was approved by the human subject committees of all collaborating institutions. Written informed consent was obtained from participants, parents or legal guardians in accordance with the Declaration of Helsinki. The Strengthening the Reporting of Observational Studies in Epidemiology (STROBE) guidelines were followed when reporting the results of the present study.

### 2.2 Data Collection

For the current study, data were retrospectively collected from the patient diary, for example, date of birth, ethnicity, date of inhibitor detection, date of ITI start, type of ITI regimen and dose defined as low (< 50 units/kg/day), intermediate (50–99 units/kg/day), or high (≥ 100 units/kg/day) in the absence of immunomodulating agents. ITI procedures including one or more immunomodulating agents were evaluated separately. If more than one regimen was used, each person was assigned the most intensive protocol for data analyzes. In addition, the historical peak inhibitor titer in each subject including all titers prior to and during ITI including the year was assessed, and categorized according to the proposal of the Future of Immune tolerance Treatment (FIT) Group [4].

The outcome of ITI was defined as success or failure according to the investigator by normal recovery and half‐life or negative Bethesda titer and normal response to FVIII therapy. The type of inhibitor response was defined as low or high based on a cut off of 5 BU/mL. Relapse of the inhibitor was defined at the discretion of the investigator.

### 2.3 Statistical Methods

Descriptive statistics were used. Continuous variables were summarized using medians and interquartile ranges (IQRs; 25th–75th percentiles). Categorical variables were reported as counts and percentages. The distributions of the different parameters were initially examined to assess their association with successful ITI using the chi‐squared test of independence. A *p*‐value < 0.05 was considered statistically significant.

A multivariable adjusted logistic regression model was constructed including parameters that were nominally significant: historical peak titer, type of *F8* variant, and age at inhibitor detection (years). In addition, ethnicity was retained as a constitutional factor commonly used in predictive modeling. The discriminatory power of the model was estimated as the concordance statistic (C‐statistic) and the equivalent area under the curve (AUC) in a receiver operator characteristic (ROC) plot. The value was calculated for the full model as well as for the model without the historical peak titer. The corresponding confidence intervals were estimated using bootstrapping.

All statistical analyzes were performed using R version 4.5.2.

## 3 Results

Characteristics of the study cohort are shown in Table 1. The entire study cohort consisted of 292 patients. Fifty‐five of these subjects had an on‐going ITI according to the treating physician, whereas 237 patients reported as, either tolerant (*n* = 173), or having failed ITI (*n* = 64) corresponding to an overall success rate in the entire study cohort of 59.2%. If those with an on‐going attempt were excluded, the rate of success was 73.0%. For low‐ (*n* = 51/53) and high‐responding patients (*n* = 122/184), the overall rate of success was 96.8% and 66.3%, respectively. Successful outcome of ITI was in 120 cases based on a normal recovery and half‐life, whereas for the remaining 53 patients, the criteria for tolerance were a negative titer and/or a normal response to FVIII therapy. Six relapses of the inhibitor were reported. All of these were exposed to another ITI attempt, 4 of whom were successfully tolerized and two were not.

**TABLE 1:** Characteristics of the study cohort.

All patients	*N* = 237 (100%)
Median age at inhibitor detection, years (IQR)	2 (1–3)
Type of inhibitor	*n* (%)
Low‐responding	53 (22.4)
High‐responding	184 (77.6)
Historical peak inhibitor titer (BU/mL)	*n* (%)
≤ 5	53 (22.4)
> 5–25	56 (23.6)
25–200	75 (31.6)
200–1000	40 (16.9)
> 1000	13 (5.5)
Ethnicity	*n* (%)
White	198 (83.5)
Hispanic	13 (5.5)
Black	7 (3.0)
Asian	8 (3.4)
Other ethnicities	11 (4.6)
Type of *F8* variant	*n* (%)
Inversion	142 (60.0)
Small ins/del	28 (11.8)
Nonsense	28 (11.8)
Missense	11 (4.6)
Large deletion	15 (6.3)
Splice site	4 (1.7)
Missing data	9 (3.8)
HLA class II	*n* (%)
HLA DRB1*1501	50 (22.2^a^)
HLA DQB1*0602	51 (22.7^a^)

^a^ The proportion of HLA class II typed patients (*n* = 225) carrying one or two of the specified HLA class II alleles.

As shown in Table 1, the median age at inhibitor detection was 2 years (IQR 1–3). This continuous variable was significantly associated with ITI outcome in our cohort, with each additional year associated with a higher risk of ITI failure (*p* = 0.007).

Among those with high‐responding inhibitors, patients with a historical peak titer between > 5 and 25 BU/mL had a better outcome (89.3%) than those with higher levels and only slightly lower that of subjects with a consistent low historical peak titer up to 5 BU/mL (96.2%) (Figure 1). In the subcohort of 75 patients with peak inhibitor titers of 25 to 200 BU/mL the success rate was 68.0%. Patients with a peak titer between 200 and 1000 BU/mL had a similar low success rate around 40% as those with a peak titer > 1000 BU/mL in our study. ITI success was highly associated with different classes of historical peak inhibitor titer (*p* < 0.0001), and a dichotomous analysis comparing subgroups with titers below and above 25 BU/mL yielded an odds ratio of 0.052 (95% CI 0.018–0.148; *p* < 0.0001).

**FIGURE 1:**
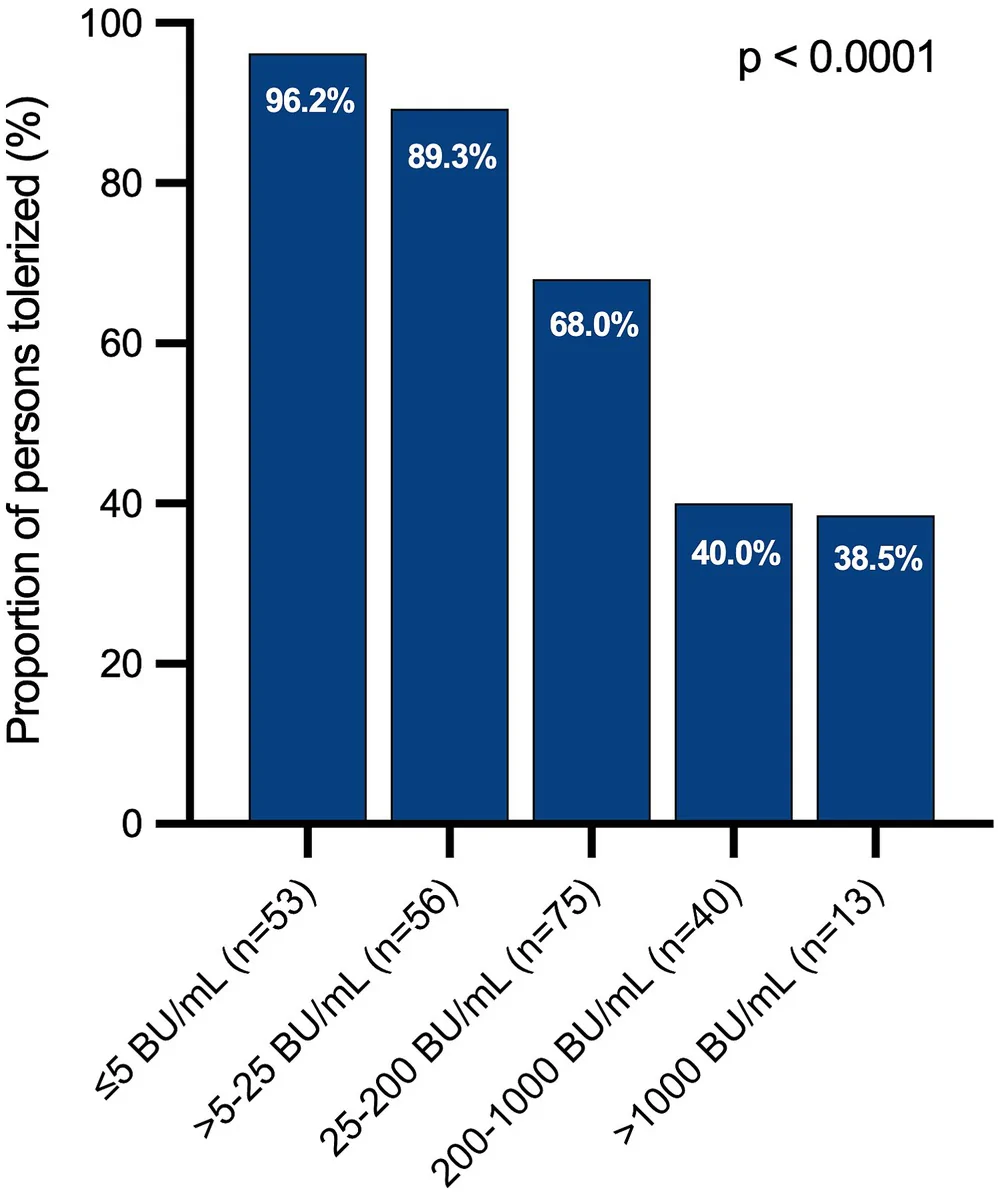
Percentage of persons tolerized by ITI stratified by the historical peak inhibitor titer (BU/mL). The *p*‐value was derived from testing the independence between ITI outcome and historical peak inhibitor titer.

Patients with large *F8* gene deletions had the lowest ITI success rate (40.0%), whereas those with missense variants had the highest success rate (90.9%) (Figure 2A). Both subgroups were, however, small. Inversions, including intron 22 inversion type 1 and 2 and intron 1 inversion, small insertions/deletions, and splice site variants exhibited similarly favorable outcomes of 75.0% to 78.2%. Nonsense variants showed an intermediate success rate of 64.3%. Although small insertions/deletions and nonsense variants located in the FVIII light chain showed numerically slightly better outcomes than those located in the heavy chain; small insertions/deletions 83.3% versus 72.2% (Figure 2B) and nonsense variants 66.7% versus 63.6% (Figure 2C), no significant correlation with the ITI outcome could be identified. The exact locations of the nonsense and small insertions/deletions along with the ITI outcome are shown in Figure 3. Overall, ITI success was significantly associated with the type of causative *F8* variant (*p* = 0.01), and a dichotomous analysis comparing subjects with large deletions to those with other *F8* variants yielded an odds ratio of 0.102 (95% CI 0.025–0.415; *p* = 0.00145).

**FIGURE 2:**
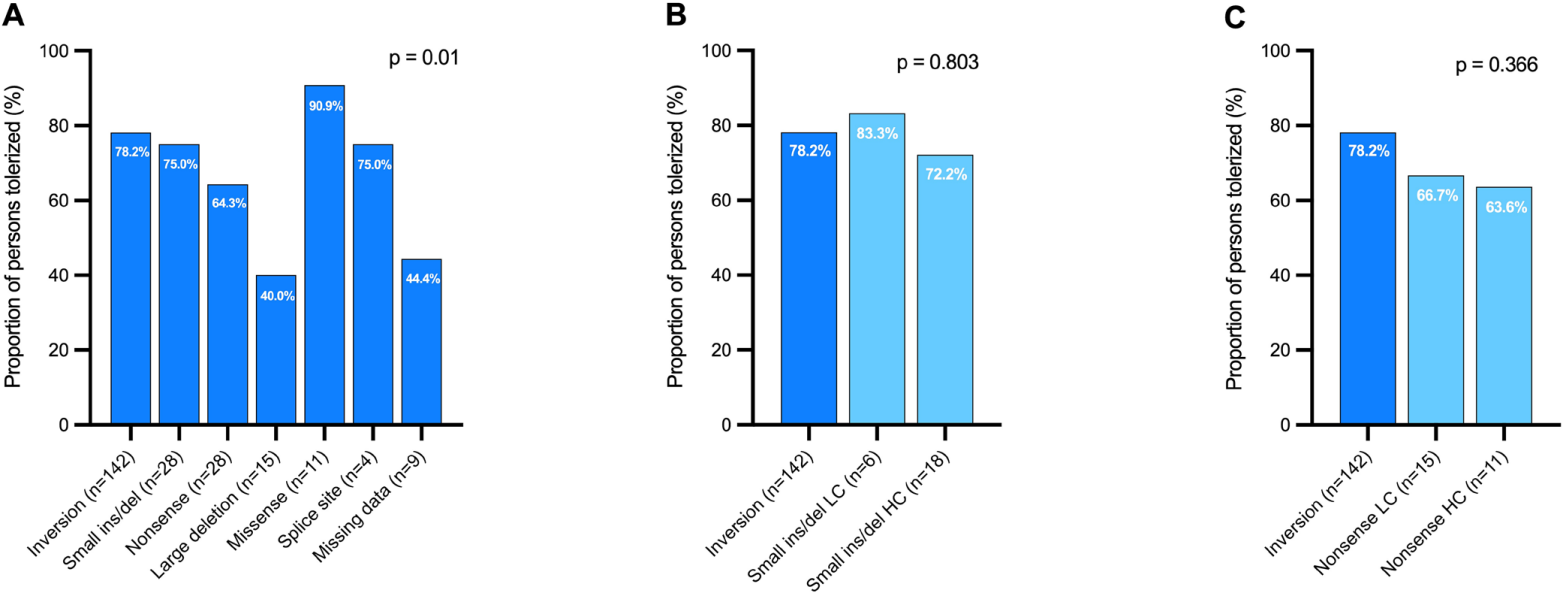
Percentage of persons tolerized by ITI stratified by the type of causative *F8* variant (A), by the location of the small ins/del (B) and nonsense variants (C) in the light chain (LC) and heavy chain (HC), respectively. The *p*‐value was derived from testing the independence between ITI outcome and the causative *F8* variant.

**FIGURE 3:**
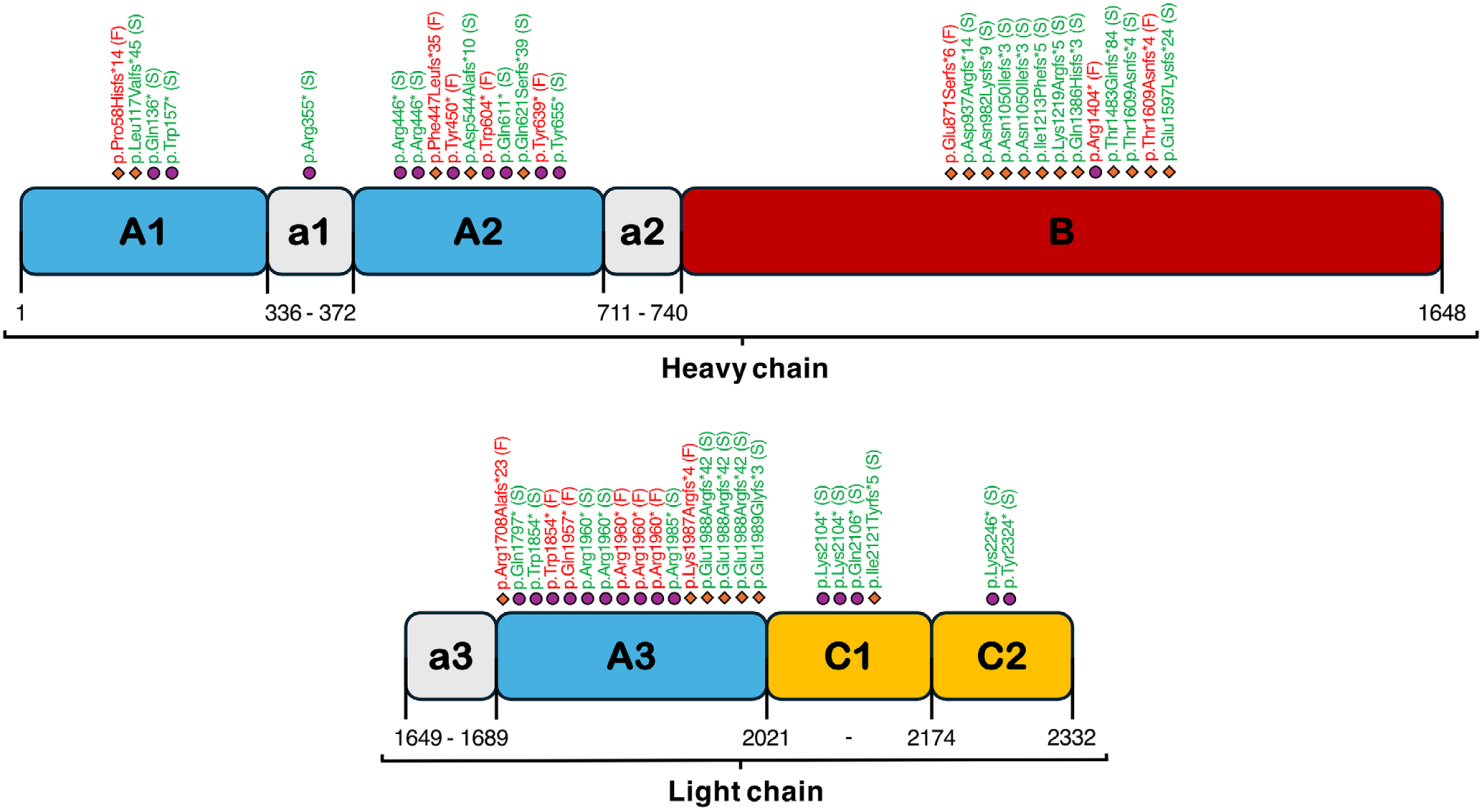
Schematic presentation of the exact locations of small ins/del causing frame shift (◇) and nonsense (◯) variants in the cohort including the ITI outcome in terms of success (S) and failure (F) marked in green and red, respectively.

Patients of Hispanic (69.2%) and Black (42.9%) ethnicity had a lower success rate than the White subcohort (74.2%), but both subgroups were small. ITI success was not associated with ethnicity (*p* = 0.48).

The HLA class II alleles HLA‐DRB1*1501 and HLA‐DQB1*0602 were strongly linked, with a *p*‐value of 4.2 × 10^−61^ in a test of independence. However, neither allele was associated with ITI outcome.

Regarding the type of concentrate used for ITI with or without the combined use of immunosuppressive agents, 116 patients were treated with recombinant and 98 with plasma‐derived concentrates with an ITI success of 81.0% and 63.3%, respectively. In 16 cases, a combination of concentrates was used and the ITI outcome in this subgroup was 87.5%. Overall, ITI success was significantly associated with the type of concentrate used (*p* = 0.004).

Regarding the ITI dose, the highest success rate (88.9%) was observed in patients receiving 50–99 units/kg/day without concomitant use of immunosuppressive agents. Patients receiving either lower or higher doses had slightly lower success rates. Immunomodulation by the use of immunosuppressants, regardless of factor VIII dose, was associated with the lowest success rate (52.8%). ITI success was significantly associated with the ITI regimen used (*p* = 0.01).

Variables exhibiting skewed distributions in relation to ITI outcome were included in multivariable‐adjusted analyzes. Reference categories were defined as follows: historical peak inhibitor titer ≤ 5 BU/mL; inversions as the causative *F8* variant; White ethnicity; low‐dose treatment regimen; and recombinant concentrate as product type. Age at inhibitor detection (years) was modeled as a continuous variable. Results are presented in Figure 4A, with corresponding estimates provided in Table 2. Factors independently associated with a lower likelihood of ITI success included large gene deletions, historical peak inhibitor titers > 25 BU/mL, and increasing age at inhibitor detection. After multivariable adjustment, neither treatment regimen nor type of factor concentrate was statistically significant and was therefore excluded from subsequent predictive modeling.

**FIGURE 4:**
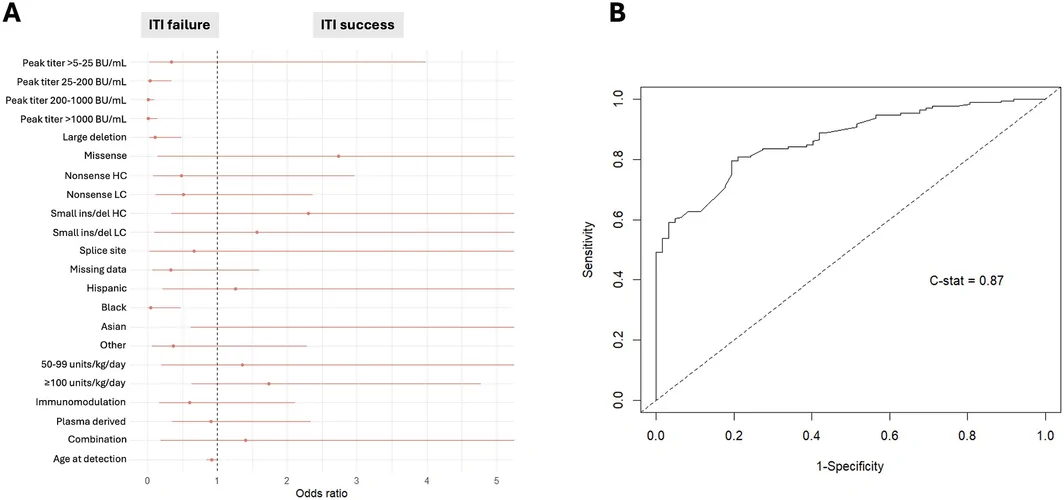
(A) Multiadjusted analyzes of predictive markers for ITI success including the variables: Historical peak titer, causative *F8* variant type, ethnicity, treatment regimen, type of concentrate and age at detection of the inhibitor. The reference category for historical peak inhibitor titer was ≤ 5 BU/mL; for causative *F8* variant, it was inversions; for ethnicity, White; for treatment regimen, < 50 units/kg/day; for type of concentrate, recombinant. Age at detection (years) was modeled as a continuous variable and each additional year was associated with a higher risk of ITI failure. The *x*‐axis was truncated at an odds ratio of 5. The exact values are provided in Table 2. (B) Receiver operating characteristic (ROC) plot for the model including historical peak titer, type of *F8* variant, ethnicity, and age at inhibitor detection. The concordance curve ranks individuals according to their combined risk factor profiles. Patients presenting with all risk factors have a very high probability of failure, whereas those with none have a very low risk. The intermediate portion of the curve reflects the full spectrum of possible risk factor combinations.

**TABLE 2:** Odds ratio (OR) estimates in the multivariable‐adjusted model for ITI success, with 95% confidence intervals (CIs) showing the lower and upper limits.

Type of variable	Estimate OR	95% CI Low	95% CI High	*p*
Historical peak inhibitor titer (BU/mL)				
≤ 5	1.00 (Ref)	—	—	—
> 5–25	0.343	0.0296	3.99	0.393
25–200	0.0378	0.00414	0.345	0.00368
200–1000	0.00989	0.000994	0.0983	0.0000816
> 1000	0.0101	0.000744	0.138	0.000567
*F8* variant				
Inversion	1.00 (Ref)	—	—	—
Large deletion	0.106	0.0234	0.482	0.00367
Missense	2.74	0.139	54	0.508
Nonsense HC	0.482	0.0781	2.97	0.431
Nonsense LC	0.516	0.113	2.36	0.394
Small ins/del HC	2.31	0.345	15.4	0.389
Small ins/del LC	1.56	0.101	24.1	0.749
Splice site	0.664	0.0271	16.3	0.802
Missing data	0.33	0.0678	1.6	0.169
Ethnicity				
White	1.00 (Ref)	—	—	—
Asian	13.7	0.619	302	0.0977
Black	0.0408	0.0035	0.477	0.0107
Hispanic	1.26	0.215	7.39	0.797
Other	0.366	0.0587	2.28	0.282
Treatment regimen				
< 50 units/kg/day	1.00 (Ref)	—	—	—
50–99 units/kg/day	1.36	0.2	9.17	0.755
≥ 100 units/kg/day	1.74	0.632	4.77	0.284
Immunomodulation	0.604	0.173	2.11	0.429
Type of product				
Recombinant	1.00 (Ref)	—	—	—
Plasma derived	0.907	0.352	2.34	0.839
Combination	1.4	0.189	10.4	0.741
Age at detection (year)	0.919	0.845	0.999	0.0468

Abbreviations: BU = Bethesda titer, Del = deletions, HC = Heavy. chain, Ins = insertions, LC = Light chain, Ref = Reference category.

The discriminatory performance of a predictive model incorporating variables significantly associated with outcome in the multivariable analysis, for example, the causative *F8* variant, historical peak inhibitor titer, and age at inhibitor detection, together with ethnicity (see Methods), was high, with a concordance statistic (C‐stat) of 0.87 (95% confidence interval [CI], 0.85–0.93) (Figure 4B). Exclusion of historical peak inhibitor titer resulted in a reduced discriminatory performance of 0.69 (95% CI, 0.64–0.80).

## 4 Discussion

This international combined cohort study evaluated markers associated with ITI success in 237 patients with severe HA and a persistent inhibitor ≥ 1 BU/mL. Our data show that peak inhibitor titer has a significant impact on ITI outcome, consistent with previously published studies [9, 20, 21, 22, 23]. However, defining precise cut‐offs for use in predictive models remains challenging. Several factors complicate this, including methodological issues for inhibitor assessment, and the time‐dependent nature of the defined peak titer, which may correlate with the frequency of measurement and treatment administered. Our findings suggest that cut‐offs of both 25 and 200 BU/mL may be considered in predictive models, with titers between 25 and 200 BU/mL indicating an intermediate probability of ITI success. The higher cut‐off of 200 BU/mL aligns with the rationale and design of the Randomized International ITI (I‐ITI) Study [7] and the recent PedNet report [14]. In contrast, the International Genetic and Clinical Determinants of the Outcome of Immune Tolerance Induction (GO‐ITI) study group proposed a lower cut‐off of 100 BU/mL for good prognosis [9, 20]. Importantly, however, our data also confirm the findings in the I‐ITI study indicating a prognostic cut‐off of 22 BU/mL and are consistent with the report by Fedewa and Kempton [22] and the cut‐off suggested by the FIT group in 2019 [4]. As expected, subjects with historical peak titers > 1000 BU had relatively poor ITI outcomes. Notably, however, more than 1/3 of these patients ultimately achieved tolerance and the outcome was similar to that of persons with inhibitor titers between 200 and 1000 BU/mL. This suggests that even patients with very high inhibitor titers may be considered for a course of ITI over a defined period taking also the treatment burden into account.

Large deletions in our cohort were associated with a significantly lower ITI success rate compared with other *F8* variants, and this is in agreement with previous reports [23, 24]. We were, however, unable to confirm either a negative impact on FVIII tolerance of small insertions/deletions, as suggested by Zuccherato et al. [23], or a positive impact, as reported by the Italian PROFIT group [25, 26]. Recent data suggest that translational readthrough mechanisms may depend on the location of nonsense variants and thereby influence inhibitor development and FVIII tolerance [17, 18]. In our study, however, we did not observe any association between ITI outcome and the location of nonsense variants or frameshift‐causing small insertions and deletions. Notably, however, we observed a nominally higher rate of ITI success in subjects with small insertions/deletions located in the light chain compared with the heavy chain (Figure 2B), and the majority of variants located in the B domain were associated with ITI success (Figure 3). Our cohort is still relatively small, but these findings are in line with the previous data on nonsense variants and may suggest that further evaluation in larger cohorts is warranted to settle whether such variants should be more finely classified to improve predictive models of ITI success.

The impact of ethnicity on inhibitor risk with higher risk in individuals of Black and Hispanic origin is largely confirmed [2]. However, the corresponding influence of ethnicity on ITI success has not been established [22, 27, 28, 29]. In our study, Black ethnicity was independently associated with poor ITI outcomes. However, given the skewed distribution of ethnicities and the small number of individuals, these findings should be interpreted with caution. We suggest, however, that ethnicity should be considered in predictive models of ITI success.

Several studies have evaluated the impact of HLA class II alleles on the risk of inhibitor development, with some consistent findings, particularly for the HLA‐DRB1*15 and HLA‐DQB1*06 loci. However, the HLA system is characterized by substantial heterogeneity and complexity, and findings should be interpreted with caution. The alleles HLA‐DRB1*1501 and HLA‐DQB1*0602 have most consistently been associated with inhibitor development, but data on ITI outcome are lacking. In our study, we observed a strong correlation between these haplotypes but found no association with ITI outcome. The type of factor used for ITI and the administered dose were not significantly associated with ITI success, but the impact of these variables was difficult to fully evaluate. Immunomodulation by the use of immunosuppressants [22], however, was associated with a low success rate (38.8%), irrespective of factor dose. This aligns with previous reports and likely reflects that the addition of immunomodulation often indicates prior ITI failures and/or a poor prognosis for other clinical reasons considered by the treating physicians. Unfortunately, detailed data on all ITI attempts for each individual were not collected, limiting further analysis.

Based on the nominally significant variables in our study, our full model demonstrated a relatively high discriminatory power of 0.87 (95% confidence interval [CI], 0.85–0.93). This value is slightly higher than that reported by Oomen et al. (C‐statistic, 0.801), who developed a predictive model incorporating cumulative FVIII exposure days at inhibitor development, peak inhibitor titer, ethnicity, and *F8* mutation type [20]. For a model to be fully useful in the clinical setting, it must not only have high discriminatory power but also include only variables that are known at the time of clinical decision‐making. This criterion is not met for the peak inhibitor titer. When the peak titer was excluded in our model, the discriminatory power declined to 0.69 (95% CI, 0.64–0.80), indicating a need for defining additional constitutional factors. Importantly, age at inhibitor detection is likely influenced in the future by the use of FVIII mimetics, since the exposure of FVIII concentrates will be significantly less frequent and distributed over a longer time period. However, the finding that each additional year of age at inhibitor detection was associated with a higher risk of ITI failure warrants consideration and further evaluation.

What about the future use of ITI in an era of new treatment options? The use of ITI has become increasingly rare, and in many centers patients are no longer offered ITI due to the treatment burden, cost, and the availability of other effective non–factor‐based treatment options. A tolerant state may, however, provide future benefits for patients. First, treatment can be administered with any FVIII concentrate in emergency situations and/or during travel, rather than relying on bypassing agents which are less effective. Second, clinical management may become less dependent on specialized care. Finally, a tolerant patient will be eligible for all future treatment options, including gene transfer and/or gene editing. Therefore, in our view, one attempt at ITI should still be considered and discussed within an informed, shared decision‐making process taking also the measured peak titer at the time into account, while awaiting improved prediction models.

Our study has both strengths and limitations. The study cohort size is relatively large, well defined, and characterized. Limitations include the retrospective design, which is inherently associated with weaknesses. In addition, we included all ITI attempts performed and defined the most intensive regimen provided, including primary and rescue treatments. This design may introduce potential bias arising from changes in preferred treatment regimens over time. However, we have attempted to address this issue through multivariable‐adjusted logistic regression modeling. Furthermore, we did not collect detailed data for each ITI course regarding the duration of treatment before a decision to stop or change therapy was made, nor the inhibitor titer at that time. The definition of success varied, potentially affecting the interpretation of the outcomes. We did not specifically ask for the peak titer before and after the start of ITI, but only for the historically highest peak. We believe this is a relevant variable for reflecting the nature of the immune response. However, from a practical point of view, only the peak titer pre‐ITI would be useful for a predictive model intended for clinical decision‐making regarding whether or not to start ITI. The pre‐ITI titer will, however, always be associated with uncertainties, since its value correlates with FVIII exposure and the timing of ITI initiation.

In conclusion, in the new era of hemophilia management, ITI has become less desirable and not pursued in all patients. However, achieving a tolerant state may still offer important advantages for long‐term health. Therefore, predicting ITI outcomes remains relevant for informed, shared decision‐making. Our findings, however, indicate the need to identify additional constitutional factors that influence the immune response and may serve as alternatives to peak inhibitor titer. For patients in whom ITI is considered, one possible approach at present may be to initiate ITI with FVIII concentrates in combination with FVIII mimetics for a limited period, allowing the inhibitor titer to be measured and used to guide clinical decision‐making. In this context, delayed inhibitor development resulting from the use of nonfactor therapies and reduced FVIII exposure may be advantageous from a venous access perspective. Nevertheless, the dosing interval will be a critical factor, and the efficacy of ITI with only one or two infusions per week using ultra‐long acting/extended half‐life FVIII concentrate warrants further evaluation.

## Author Contributions

**Marcus Fager Ferrari:** data curation, formal analysis, investigation, visualization, writing – review and editing. **Josefin Roslund:** data curation, investigation, writing – review and editing. **Eric Manderstedt:** formal analysis, visualization, writing – review and editing. **Erik Berntorp:** conceptualization, writing – review and editing. **Jan Astermark:** conceptualization, data curation, formal analysis, investigation, visualization, funding acquisition, writing – original draft, writing – review and editing.

## Funding

The study was financed by grants from the Swedish Research Council and the Swedish state under the agreement between the Swedish government and the county councils, the ALF‐agreement (project grant 2022‐0205) and Skane University Hospital. The Hemophilia Inhibitor Genetics Study was supported by an investigator‐initiated grant from Baxter BioScience, and in part with federal funds from the National Institutes of Health, National Cancer Institute, N01‐CO‐12400.

## Ethics Statement

Ethical approval for all patients attending the Malmö hemophilia center was obtained from the Swedish Ethical Review Authority. Malmö and Hemophilia Inhibitor Genetics Study Investigators was approved by the human subject committees of all collaborating institutions.

## Consent

Written informed consent was obtained from participants, parents or legal guardians in accordance with the Declaration of Helsinki.

## Conflicts of Interest

M.F.F. has received speakers fee from AstraZeneca and LEO Pharma. J.R. and E.M. have no conflicts of interest. E.B. has received research grants from Sobi, Sanofi, CSL Behring and Bayer. Speakers fee and consultant for Bayer, Sobi, Octapharma, Novo Nordisk, Bayer, CSL Behring, Roche, Takeda/Shire. J.A. has received research grants from Sobi, Sanofi and CSL Behring. Speakers fee and consultant for Sobi, Octapharma, Novo Nordisk, Pfizer, Bayer, CSL Behring, Takeda/Shire, and BioMarin.

## Data Availability

The data that support the findings of this study are available on request from the corresponding author. The data are not publicly available due to privacy or ethical restrictions.

## References

[1] E. Berntorp and A. D. Shapiro , “Modern Haemophilia Care,” Lancet 379, no. 9824 (2012): 1447–1456. 10.1016/S0140-6736(11)61139-2 22456059

[2] J. Astermark , “FVIII Inhibitors: Pathogenesis and Avoidance,” Blood 125, no. 13 (2015): 2045–2051. 10.1182/blood-2014-08-535328 PMC4416939 25712994

[3] C. L. Eckhardt , J. I. Loomans , A. S. van Velzen , et al., “Inhibitor Development and Mortality in Non‐Severe Hemophilia A,” Journal of Thrombosis and Haemostasis 13 (2015): 1217–1225. 10.1111/jth.12990 25912309

[4] M. Carcao , C. Escuriola Ettinghausen , E. Santagostino , et al., “The Changing Face of Immune Tolerance Induction in Haemophilia A With the Advent of Emicizumab,” Haemophilia 25 (2019): 676–684. 10.1111/hae.13762 PMC6850066 31033112

[5] D. P. Hart , J. Alamelu , N. Bhatnagar , et al., “Immune Tolerance Induction in Severe Haemophilia A: A UKHCDO Inhibitor and Paediatric Working Party Consensus Update,” Haemophilia 27 (2021): 932–937. 10.1111/hae.14381 34403546

[6] K. Holstein , S. Le Quellec , R. Klamroth , et al., “Immune Tolerance Induction in the Era of Emicizumab – Still the First Choice for Patients With Haemophilia A and Inhibitors?,” Haemophilia 28 (2022): 215–222. 10.1111/hae.14470 34918839

[7] C. R. Hay , D. M. DiMichele , and International Immune Tolerance Study , “The Principal Results of the International Immune Tolerance Study: A Randomized Dose Comparison,” Blood 119, no. 6 (2012): 1335–1344. 10.1182/blood-2011-08-369132 22101900

[8] H. H. Brackmann , G. C. White, 2nd , E. Berntorp , H. H. Brackmann , T. Andersen , and C. Escuriola‐Ettingshausen , “Immune Tolerance Induction: What Have We Learned Over Time?,” Haemophilia 24, no. Suppl 3 (2018): 3–14. 10.1111/hae.13445 29543371

[9] I. Oomen , R. M. Camelo , S. M. Rezende , et al., “Determinants of Successful Immune Tolerance Induction in Hemophilia A: Systematic Review and Meta‐Analysis,” Research and Practice in Thrombosis and Haemostasis 7, no. 1 (2022): 100020. 10.1016/j.rpth.2022.100020 PMC9986104 36891524

[10] P. Chowdary , M. Carcao , G. Kenet , and S. W. Pipe , “Haemophilia,” Lancet 405, no. 10480 (2025): 736–750. 10.1016/S0140-6736(24)02139-1 40023652

[11] R. W. Herzog , R. Kaczmarek , and K. A. High , “Gene Therapy for Hemophilia ‐ From Basic Science to First Approvals of “One‐And‐Done” Therapies,” Molecular Therapy 33, no. 5 (2025): 2015–2034. 10.1016/j.ymthe.2025.03.043 PMC12126786 40156189

[12] J. Oldenburg , J. K. Picard , R. Schwaab , H. H. Brackmann , E. G. D. Tuddenham , and E. Simpson , “HLA Genotype of Patients With Severe Haemophilia A due to Intron 22 Inversion With and Without Inhibitors of Factor VIII,” Thrombosis and Haemostasis 77, no. 2 (1997): 238–242. 9157573

[13] C. R. Hay , W. Ollier , L. Pepper , et al., “HLA Class II Profile: A Weak Determinant of Factor VIII Inhibitor Development in Severe Haemophilia A. UKHCDO Inhibitor Working Party,” Thrombosis and Haemostasis 77, no. 2 (1997): 234–237. 9157572

[14] H. Pergantou , I. Varela , O. Moraloglou , et al., “Impact of HLA Alleles and Cytokine Polymorphisms on Inhibitors Development in Children With Severe Haemophilia A,” Haemophilia 19, no. 5 (2013): 706–710. 10.1111/hae.12168 23607306

[15] S. Lessard , C. He , D. K. Rajpal , et al., “Genome‐Wide Association Study and Gene‐Based Analysis of Participants With Hemophilia A and Inhibitors in the My Life, Our Future Research Repository,” Frontiers in Medicine 9 (2022): 903838. 10.3389/fmed.2022.903838 PMC9260508 35814780

[16] V. P. Diego , B. W. Luu , M. A. Almeida , et al., “Disentangling Effects of the DR and DQ Isomers Encoded by the HLA Class II Haplotype DRB1*15:01/DQB1*06:02 to Help Establish the True Risk Allele for FVIII Inhibitor Development in Hemophilia A,” Frontiers in Genetics 16 (2025): 1506862. 10.3389/fgene.2025.1506862 PMC12016221 40270541

[17] M. F. Testa , S. Lombardi , F. Bernardi , et al., “Translational Readthrough at F8 Nonsense Variants in the Factor VIII B Domain Contributes to Residual Expression and Lowers Inhibitor Association,” Haematologica 108, no. 2 (2023): 472–482. 10.3324/haematol.2022.281279 PMC9890017 35924581

[18] M. F. Testa , “A Novel Genetic Classification of Inhibitor Risk for F8 Nonsense Mutations Based on Immunogenic Profiling of Ribosomal Readthrough in EAHAD Database,” Haemophilia 32, no. 1 (2026): 14.

[19] J. Astermark , S. M. Donfield , E. D. Gomperts , et al., “The Polygenic Nature of Inhibitors in Hemophilia A: Results From the Hemophilia Inhibitor Genetics Study (HIGS) Combined Cohort,” Blood 121, no. 8 (2013): 1446–1454. 10.1182/blood-2012-06-434803 PMC3578958 23223434

[20] I. Oomen , A. Abdi , R. M. Camelo , et al., “Prediction of the Chance of Successful Immune Tolerance Induction in Persons With Severe Hemophilia A and Inhibitors: A Clinical Prediction Model,” Research and Practice in Thrombosis and Hemostasis 8, no. 7 (2024): 102580. 10.1016/j.rpth.2024.102580 PMC11570954 39558913

[21] M. Carcao , C. Königs , N. G. Andersson , et al., “Predictors of Immune Tolerance Induction Success in 231 Children With Severe Hemophilia A With High‐Titer Inhibitors—Lessons Learned From the PedNet Prospective Cohort Study,” Journal of Thrombosis and Haemostasis 23, no. 10 (2025): 3134–3147. 10.1016/j.jtha.2025.07.010 40706963

[22] S. A. Fedewa and C. L. Kempton , “Race and Ethnicity and the Success of Immune Tolerance Induction Among People With Severe Haemophilia A in the United States,” Haemophilia 30, no. 3 (2024): 628–637. 10.1111/hae.14980 38462837

[23] L. W. Zuccherato , R. P. Souza , R. M. Camelo , et al., “Large Deletions and Small Insertions and Deletions in the Factor VIII Gene Predict Unfavorable Immune Tolerance Induction Outcome in People With Severe Hemophilia A and High‐Responding Inhibitors,” Thrombosis Research 242 (2024): 109115. 10.1016/j.thromres.2024.109115 39186847

[24] I. Oomen , A. Abdi , L. Broer , et al., “Large Deletions in the F8 Gene Predict Immune Tolerance Induction Failure in People With Severe Hemophilia A,” Research and Practice in Thrombosis and Haemostasis 9, no. 7 (2025): 103212. 10.1016/j.rpth.2025.103212 PMC12616064 41246453

[25] A. Coppola , M. Margaglione , E. Santagostino , et al., “Factor VIII Gene (F8) Mutations as Predictors of Outcome in Immune Tolerance Induction of Hemophilia A Patients With High‐Responding Inhibitors,” Journal of Thrombosis and Haemostasis 7, no. 11 (2009): 1809–1815. 10.1111/j.1538-7836.2009.03615.x 19740093

[26] G. Di Minno , A. Coppola , M. Margaglione , et al., “Predictors of Inhibitor Eradication by Primary Immune Tolerance Induction in Severe Haemophilia A With High Responding Inhibitors,” Haemophilia 28, no. 1 (2022): 55–64. 10.1111/hae.14431 34727394

[27] M. U. Callaghan , M. Rajpurkar , M. Chitlur , I. Warrier , and J. Lusher , “Immune Tolerance Induction in 31 Children With Haemophilia A: Is ITI Less Successful in African Americans?,” Haemophilia 17, no. 3 (2011): 483–489. 10.1111/j.1365-2516.2010.02429.x 21118335

[28] R. P. N. Kruse‐Jarres and C. A. Leissinger , “The Role of Race and Ethnicity in the Clinical Outcomes of Severe Hemophilia A Patients With Inhibitors,” Blood 110, no. 11 (2007): 1163.

[29] D. M. DiMichele , B. L. Kroner , and North American Immune Tolerance Study G , “The North American Immune Tolerance Registry: Practices, Outcomes, Outcome Predictors,” Thrombosis and Haemostasis 87, no. 1 (2002): 52–57. 11848456

